# Early malnutrition results in long-lasting impairments in pattern-separation for overlapping novel object and novel location memories and reduced hippocampal neurogenesis

**DOI:** 10.1038/srep21275

**Published:** 2016-02-17

**Authors:** Georgina Pérez-García, Omar Guzmán-Quevedo, Raquel Da Silva Aragão, Francisco Bolaños-Jiménez

**Affiliations:** 1 INRA, UMR1280 Physiologie des Adaptations Nutritionnelles, Université de Nantes, Nantes Atlantique Université, 44096, Nantes, France

## Abstract

Numerous epidemiological studies indicate that malnutrition during *in utero* development and/or childhood induces long-lasting learning disabilities and enhanced susceptibility to develop psychiatric disorders. However, animal studies aimed to address this question have yielded inconsistent results due to the use of learning tasks involving negative or positive reinforces that interfere with the enduring changes in emotional reactivity and motivation produced by *in utero* and neonatal malnutrition. Consequently, the mechanisms underlying the learning deficits associated with malnutrition in early life remain unknown. Here we implemented a behavioural paradigm based on the combination of the novel object recognition and the novel object location tasks to define the impact of early protein-restriction on the behavioural, cellular and molecular basis of memory processing. Adult rats born to dams fed a low-protein diet during pregnancy and lactation, exhibited impaired encoding and consolidation of memory resulting from impaired pattern separation. This learning deficit was associated with reduced production of newly born hippocampal neurons and down regulation of BDNF gene expression. These data sustain the existence of a causal relationship between early malnutrition and impaired learning in adulthood and show that decreased adult neurogenesis is associated to the cognitive deficits induced by childhood exposure to poor nutrition.

A large amount of epidemiological data indicates that a deficient provision of nutrients during *in utero* development and/or childhood, results in long-lasting impaired attention and learning, decreased IQ scores and reduced visuospatial working memory^1^,^2^,^3^,^4^ leading to academic underachievement and lower productive capacity in adult life. Nevertheless, since most of these data concern populations from developing countries, it has been difficult to clearly establish whether the observed cognitive deficits are due to social and economic factors inherent to poverty, like poor psychological stimulation and cultural deprivation, rather than to malnutrition *per se.* Thus, while correlational studies conducted in Jamaica and Barbados have found decreased cognitive capacities in adolescents and adults that had experienced protein-calorie malnutrition in infancy^5^,^6^, no defects in learning skills were observed in a cohort of people born in the Netherlands during the Dutch famine^7^,^8^. Interventional studies have also shown that cognitive stimulation during early childhood is more effective than nutritional supplementation to reverse the deleterious cognitive effects of malnutrition^5^. Similarly, impaired^9^,^10^,^11^,^12^ or normal^13^,^14^,^15^ learning capacities have been reported in early malnourished rats. These latter conflicting results might be explained by the differences in the type and severity of the nutritional insult^4^. Yet, the most important source of bias in all the experimental studies performed to date to evaluate the cognitive capacities of previously malnourished animals is the fact that they have relied on the use of learning paradigms that contain a stressful component or utilise food as positive reinforcer^16^. This is of particular concern because it is well established that early malnutrition leads to increased anxiety and sensitivity to aversive or painful stimuli^17^,^18^ and to enhanced motivation for food^19^,^20^.

Regarding the mechanisms that might underlie the cognitive deficits of early malnutrition, studies in rats have shown that maternal protein or calorie restriction leads to decreased number of neurons, spines, synapses, and dendritic arborisations in the dentate gyrus of the offspring^21^,^22^. However, it is difficult to establish a causal link between these perturbations and impaired cognition in the light of what we know about the neurobiological basis of learning. Indeed, though the contribution of adult hippocampal neurogenesis to the process of learning and memory is well established^23^,^24^, only few studies have analysed how malnutrition during early life impacts on cell proliferation and differentiation in brain at adulthood^25^,^26^,^27^,^28^. Moreover, none of the studies performed to date has addressed the question of which component of the learning process (encoding, consolidation, retrieval), is affected by early malnutrition.

Here we implemented a learning paradigm based on the combination of the Novel Object Recognition (NOR) and the Novel Object Location (NOL) tasks to define the impact of early protein-restriction on memory formation and consolidation and to investigate the cellular and molecular mechanisms underpinning the learning disabilities of early malnourished individuals. These tests relay on the animal’s innate preference for novelty such that learning is not driven by external positive or negative motivational factors, like food reward or stressful and painful stimuli, avoiding bias in the interpretation of behavioural results associated with the effects of malnutrition on feeding and significantly limiting confounding biases due to impaired emotional reactivity.

## Results

### Effects of early protein-restriction on learning

**Figure 1:**
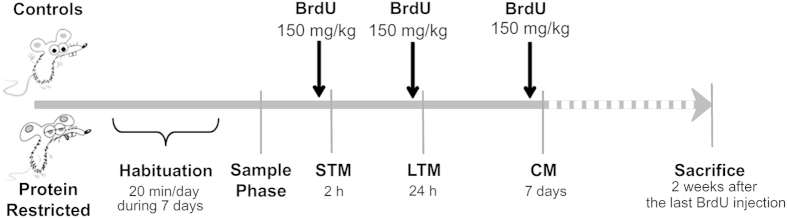
Schematic diagram giving an overall picture of the sequential order of the memory tasks and BrdU injections. The administration of BrdU was done one hour before each learning task and the end of the sample phase was considered as time zero. Given that we expected an increased production of neurons in response to learning, this administration paradigm was chosen to label a maximum of proliferating cells. Animals were allowed 7 min to explore the objects in all the sessions. STM = Short Term Memory; LTM = Long Term Memory; CM = Consolidated Memory.

**Figure 2:**
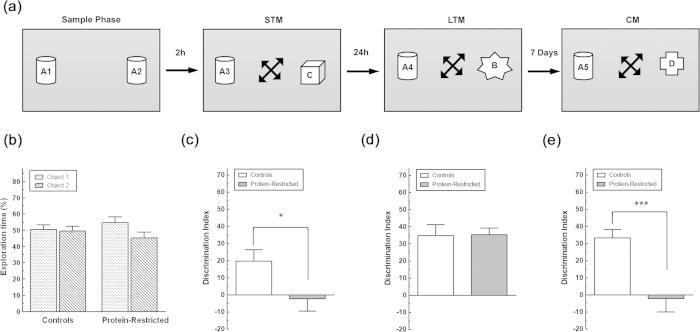
Impact of maternal protein-restriction on the learning capacity of the offspring at adulthood. (**a**) Drawing illustrating the sample phase and the learning sessions of the combined novel object recognition and novel object location memory test. Arrows inside the boxes indicate that both the familiar and the novel object were placed at random in a different position they have occupied in the previous test session. Arrows between the diagrams indicate the time interval between the sample phase and the NOR tasks. (**b**) Time spent exploring the objects during the sample session by control and PR rats. Exploration time for each object is expressed as percentage of the total time rats spent exploring the two objects. (**c**–**e**), Discrimination index during the choice phase of the NOR sessions. Note that adult animals born to protein-restricted dams show a delay acquisition and impaired consolidation of the object trace. *p < 0.05; ***p < 0.001 as determined by two-way ANOVA for repeated measures followed by Bonferroni’s multiple comparison test with n = 11 animals per group.

The novel object recognition task is one of the most widely used models for the study of the neurobiological basis of learning and memory in rodents. In the first version of this behavioural test^29^, animals are briefly exposed to two identical objects and memory is evaluated in a second test trial by exchanging one of the familiar objects for a novel one. During the test, preferential exploration of the novel versus the familiar object provides a measure of recognition memory. If the two objects initially explored are presented during the test session, but with one of the objects placed at a different spatial location that during training, the displaced familiar object is explored to the same extent as a novel object^30^, providing a measure of spatial memory. In the present study, we used a combination of these two protocols to evaluate the three basic processes of memory: enconding/consolidation/retrieval. We also aimed to model pattern separation, a key component of memory processing. We reasoned that the repeated exposure to a familiar object at different places within a familiar context along with the presentation of a novel object, would reinforce the memory updating component of the test, allowing past episodes of object recognition to be reactivated, interleaved with new experience and consolidated and, at the same time, would allow to evaluate the capacity of the animal to discriminate and encode into distinct memory representations spatial and recognition information that is very similar and easily confused for one another (pattern separation). Moreover, in order to determine whether the learning disabilities of early malnourished individuals are due to impaired neurogenesis, animals received one i.p. injection of BrdU one hour before each learning test. The general experimental design is illustrated in Fig. 1 whereas the learning protocol is shown in Fig. 2a.

Control and PR rats spent the same time exploring the two objects during the sample session (Fig. 2b). However, when both groups were exposed 2 h later to a copy of the objects presented in the sample session and a novel object (short-term memory), control animals, but not PR rats, exhibited a clear preference to explore the novel object as shown by the significant interaction between maternal diet and NOR session (F_(2,40)_ = 4.950, P = 0.012, Fig. 2c). Two-way repeated measures ANOVA also indicated that there was a significant main effect of maternal diet (F_(1,20)_ = 10.26, P = 0.0045), and NOR session (F_(2,40)_ = 11.24, P = 0.0001) on the capacity to discriminate the novel from the familiar object. Control rats continued to exhibit a preference to explore the novel object in relation to the familiar one 24 h (long-term memory) and 7 days after the training session (consolidated memory, Fig. 2d,e). In contrast, although PR rats exhibited an identical discrimination index to that of control rats during the 24 h post-sample session (Fig. 2d), seven days after the sample phase they were again unable to distinguish between the novel and the familiar objects (Fig. 2e).

**Figure 3:**
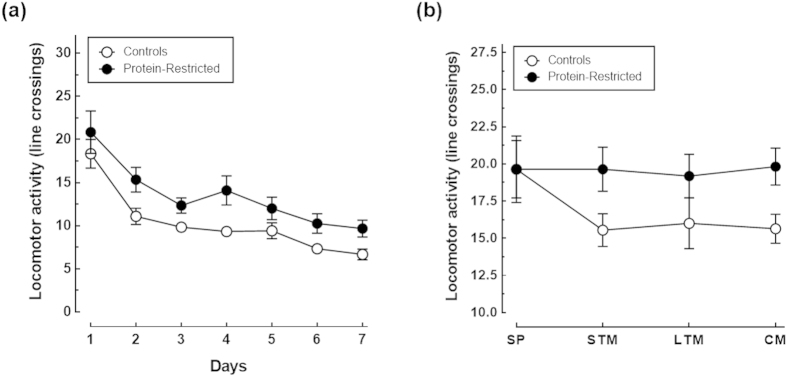
Impact of early protein-restriction on locomotor activity during the habituation (**a**) and NOR (**b**) sessions. Data correspond to the mean ± S.E.M of line crossings with n = 12 animals per group. There were not significant differences in locomotor activity between the groups as assessed by two-way repeated measures ANOVA followed by Bonferroni’s multiple comparison tests.

**Figure 4:**
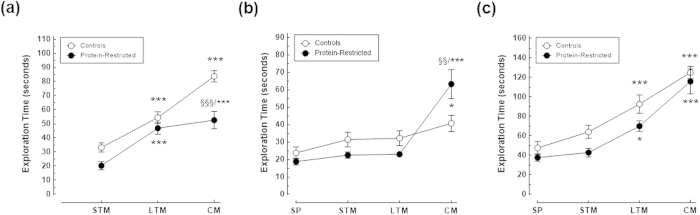
Impact of maternal protein-restriction on the exploratory activity of the offspring at adulthood. Total amount of time spent exploring the novel (**a)** and the familiar (**b**) objects and total time spent exploring both objects (**c**), by control and protein-restricted animals during the sample phase and NOR tasks. The time spent by each rat exploring the objects increased progressively from one test trial to the other but the total amount of exploration time did not differ between the groups. Note, however, that PR rats spent more time exploring the familiar object than the novel one during the consolidated memory (CM) session and, conversely, control rats spent more time exploring the novel object than the familiar one. *p < 0.05; ***p < 0.001 compared to exploration time during the sample phase (SP) or the short-term memory session (STM) of the same experimental group (Dunnett’s multiple comparisons test). ^§§^p < 0.01; ^§§§^p < 0.001compared to exploration time of control animals during the same NOR session (Bonferroni’s multiple comparison tests).

Importantly, control and PR animals exhibited identical levels of locomotor activity as indicated by the similar number of line crossings during the habituation and test sessions (Fig. 3). Actually, there was no a significant interaction between maternal diet and session on locomotor activity neither during habituation (F_(6,132)_ = 0.3052, P = 0.9333), nor during the NOR sessions (F_(3,60)_ = 1.224, P = 0.3089). Moreover, both control and PR rats spent more time exploring the novel object during the LTM and CM tests sessions than during the STM session as determined by one-way ANOVA for repeated measures (Fig. 4a). Further two-way ANOVA analysis followed by Bonferroni’s post-test showed that control animals explored more time the novel object than PR animals but only during the evaluation of consolidated memory (Fig. 4a), with a main effect of maternal diet (F_(2,38)_ = 19.02, P = 0.0003), and of NOR session (F_(2,38)_ = 58.14, P < 0.0001), and a significant interaction between these two factors (F_(2,38)_ = 4.90, P = 0.0128). A similar exploration pattern was observed when the time spent exploring the familiar object across the NOR sessions was analysed. That is, control and PR rats spent more time exploring the familiar object during the learning sessions than during the sample phase though these differences reached only statistical significance during the CM session as assessed by post-hoc analysis using one-way ANOVA and Dunnett’s multiple comparisons test (Fig. 4b). However, PR animals spent more time than their control counterparts exploring the familiar object during the CM test session as revealed by two-way ANOVA (maternal diet x NOR session interaction F_(3,60)_ = 6.314, P = 0.0009; main effect of maternal diet F_(1,20)_ = 0.0021, P = 0.9637; main effect of NOR session F_(3,60)_ = 20.19, P < 0.0001). Of note, the total time control and PR rats spent exploring objects increased for one test session to the other (Fig. 4c). However, no differences were found between the groups in total exploration time neither during the sample phase nor during the NOR sessions (maternal diet x NOR session interaction F_(3,60)_ = 0.4845, P = 0.6943). Collectively, these results indicate that: 1) control rats kept in memory the initial spatial location of the familiar object and that they can distinguish it from the novel one at least during seven days after a single sample session; 2) early protein-restriction does not interfere with the locomotor activity of the animals; 3) the impaired learning performance of PR rats during the first and third test sessions is not due to an attention deficit but likely results from impaired memory consolidation and/or reconsolidation.

### Effects of early protein-restriction on adult neurogenesis

**Figure 5:**
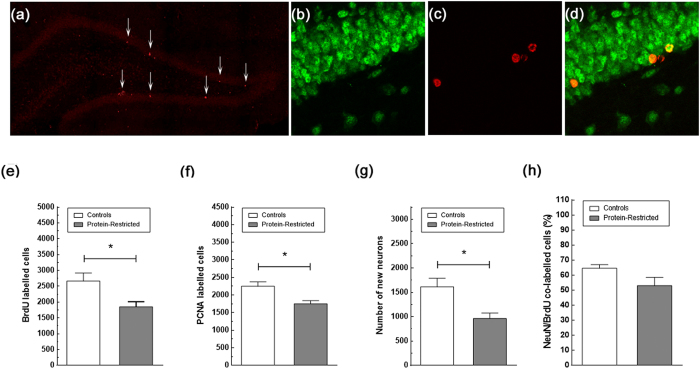
Impact of early protein-restriction on hippocampal neurogenesis at adulthood. Representative confocal images of the dentate gyrus stained for PCNA (**a**), NeuN (**b**), BrdU (**c**), and double stained for BrdU and the neuronal marker NeuN (**d**) in controls rats. Arrows in (**a**) point to PCNA immuno-positive cells. Cells double-labeled with BrdU and NeuN were counted as new neurons. Data correspond to the mean ± SEM of the total number of BrdU (**e**), PCNA (**f**) and double labeled cells (**g**), and to the percentage of BrdU/NeuN stained cells in relation to the total number of BrdU positive cells (**h**). Note that, though naïve PR rats exhibit a reduced number of BrdU-labeled cells co-expressing the neuronal marker NeuN and, therefore, of new neurons, there are no differences between control and PR rats in the proportion of BrdU/NeuN-labeled cells *p < 0.05, Student’s t-test with n = 5–8 animals per group.

Learning increases the production of new hippocampal neurons^31^,^32^,^33^ and these new generated neurons are involved in memory processing notably in pattern separation a key component of memory consolidation/reconsolidation. Therefore, we tested whether the learning deficits of PR rats could be explained by an impaired neurogenesis response. To this end, we first evaluated by BrdU labelling the number of new generated hippocampal cells under naive conditions. We found a reduction in the number of BrdU-positive cells between control and PR rats that was statistically significant by Student’s t-test analysis (C = 2664 ± 255; PR = 1850 ± 164, P = 0,027 Fig. 5e), suggesting that early protein-restriction decreases the capacity of the hippocampus to produce new cells. However, given that BrdU staining was analyzed two weeks after the last injection of BrdU, and therefore providing an estimation of cell survival, the question remains as to whether early protein-restriction reduces cell proliferation and/or cell survival. To clearly ascertain this point, a different set of slices from each experimental group was immuno-labelled with an antibody against PCNA. In contrast to BrdU, which is incorporated into DNA during the S-phase of the cell cycle and, thus, is a marker of both cell proliferation and cell survival, PCNA is endogenously expressed by mitotic cells during the G1- and S-phases of the cell cycle such that its immunological visualisation gives an instantaneous picture of cell proliferation. In agreement with the results of BrdU staining, naïve PR rats exhibited a decreased number of immunopositive PCNA cells compared with naive control animals (C = 2251 ± 122; PR = 1753 ± 87, P = 0.0115, Student’s t-test, Fig. 5f) indicating that protein-restriction induces a long-lasting reduction in both cell proliferation and cell survival. We next determined the effects of perinatal undernutrition on the neuronal differentiation process of the new generated cells using as criteria to identify the new neurons the number of BrdU-immunopositive cells co-expressing NeuN, a marker for mature neurons. In line with the results of BrdU and PCNA staining, there was a significant decrease in the number of new neurons in PR rats compared with controls (C = 1615 ± 174 vs PR = 962 ± 110, P = 0.0148, Student’s t-test, Fig. 5g). However, when the number of co-labelled cells was expressed as percentage of the total number of BrdU-stained cells, there were no differences between control and PR rats (C = 64.61 ± 2.29% vs PR = 54.78 ± 4.85%, P = 0.096, Student’s t-test, Fig. 5h). Together with the results of PCNA staining, this observation indicates that early protein-restriction induces a long-lasting impairment in the proliferation but not the survival or the neuronal differentiation process of hippocampal stem cells.

**Figure 6:**
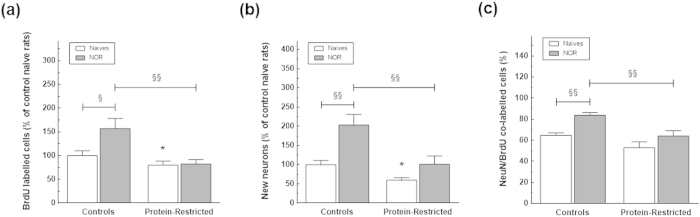
Impact of early protein-restriction on neurogenesis in response to learning. Data in (**a**) and (**b**) correspond, respectively, to the number of BrdU cells and new neurons expressed as percentage of the mean BrdU and BrdU/NeuN counts for the control group whereas the values in (**c**) correspond to the percentage of BrdU/NeuN stained cells in relation to the total number of BrdU positive cells within each group. *p < 0.05 compared with control naïve animals (Student’s t-test). ^§^p < 0.05; ^§§^p < 0.01 (two-way ANOVA followed by Bonferroni’s multiple comparison tests).

The analysis of the effects of protein-restriction on neurogenesis in response to learning, showed that NOR increased significantly the number of BrdU-labelled cells in control rats (Fig. 6a). In contrast, NOR-trained PR rats exhibited the same number of immunopositive BrdU cells that their naïve counterparts. Two-way ANOVA analysis revealed a significant interaction between maternal diet and NOR (F_(1,17)_ = 5.71, P = 0.0287), as well as a main effect of NOR (F_(1,17)_ = 6.65, P = 0.0195) and maternal diet (F_(1,17)_ = 14.80, P = 0.0013). In addition, NOR stimulated neurogenesis in control rats as indicated by the increased number of new neurons (maternal diet effect F_(1,18)_ = 13.70, P = 0.0016; NOR effect F_(1,18)_ = 14.18, P = 0.0014, maternal diet x NOR interaction F_(1,18)_ = 2.58, P = 0.1258, Fig. 6b) and proportion of BrdU/NeuN labelled cells (maternal diet effect F_(1,18)_ = 16.75, P = 0.0007; NOR effect F_(1,18)_ = 15.68, P = 0.0009, maternal diet x NOR interaction F_(1,18)_ = 1.07, P = 0.3154, Fig. 6c) in NOR-trained animals versus naive rats. In contrast to control animals, trained and naive PR rats exhibited a similar number (Fig. 6b) and percentage (Fig. 6c) of BrdU cells showing a neuronal phenotype.

### Effects of early protein-restriction on BDNF and Zif 268 gene expression

**Figure 7:**
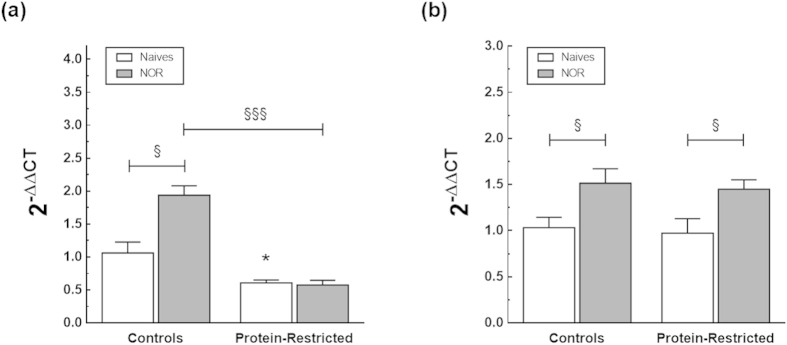
Iact of early protein-restriction on hippocampal BDNF (**a**) and Zif268 (**b**) gene expression. The hippocampus was dissected from adult rats born to dams fed a control or a low-protein diet during gestation and lactation that were either undisturbed in their home cages or evaluated for Short Term Memory using the NOR task. Animals were sacrificed 2 h after the end of the learning session and the levels of the transcripts determined by real-time PCR. Values illustrate the relative abundance of each transcript in relation to those of 18S amplified within the same sample and under the same experimental conditions. *p < 0.05 compared with control naïve animals (Student’s t-test). ^§^p < 0.05; ^§§§^p < 0.001 (two-way ANOVA followed by Bonferroni’s multiple comparison tests). The number of animals within each group ranged from 4 to 6.

Memory consolidation/reconsolidation of pattern-separated memories has been shown to be modulated by BDNF and Zif268^34^,^35^. We therefore asked whether early protein-restriction alters in the long term the expression of these factors. To this end, the hippocampal levels of mRNAs encoding BDNF and Zif268 were quantified by real-time RT-PCR in a different set of control and PR animals that were evaluated for short-term memory and sacrificed 2 h after the end of the NOR task. Under naive conditions, the mRNA levels of BDNF were significantly decreased in the hippocampus of the offspring born to protein-restricted dams compared to their control counterparts (2^−ΔΔCt^ value controls =1.060 ± 0.166 vs PR = 0.6080 ± 0.04, P = 0.039, Student’s t-test, Fig. 7a). In contrast, there were no significant differences between control and PR rats in the mRNA expression of Zif268 (2^−ΔΔCt^ value controls = 1.032 ± 0.127 vs PR = 1.088 ± 0.173, P = 0.789). Moreover, whereas the gene expression of both BDNF and Zif268 increased significantly in response to NOR in control rats (Fig. 7b), only the mRNA levels of Zif268 were enhanced in PR animals after NOR exposure. The results of the two-way ANOVA analysis for the combined effects of protein-restriction and NOR on gene expression are as follows: maternal diet effect for BDNF expression F_(1,16)_ = 53.24, P < 0.0001; maternal diet effect for ZIF268 expression_(1,18)_ = 1.14, P = 0.298; main effect of NOR for BDNF expression F_(1,16)_ = 11.57, P = 0.0036; main effect of NOR for ZIF268 expression F_(1,18)_ = 19.30 P = 0.0004; maternal diet x NOR interaction for BDNF expression F_(1,16)_ = 13.39, P = 0.0021; maternal diet x NOR interaction for ZIF268 expression F_(1,18)_ = 0.29, P = 0.5970).

## Discussion

In spite of extensive research, a clear cause-effect relationship between early malnutrition and learning disabilities later in life has not been established. We also ignore what might be the cellular and molecular mechanisms underpinning the cognitive deficits in previously malnourished individuals and there is a complete lack of information about how undernutrition during pregnancy and childhood impacts on memory processing in adolescence and adulthood. In the present study we used a behavioural paradigm based on the combination of the novel object recognition and novel object location tasks to identify which component of the memory process - enconding/acquisition, retrieval or consolidation/reconsolidation–is affected by malnutrition during *in utero* development and neonatal life. Notably, animals were exposed to three consecutive test sessions at different retention delays after the sample phase (2 h, 24 h and 7 days). For each test session one copy of the objects presented during the sample phase was introduced in the test arena along with a novel object of different shape, height and colour and both the novel and the familiar objects were counterbalanced and placed in a different location relative to the position they occupied in the previous test session. Since one of the objectives of our study was to determine if the learning disabilities of early malnourished individuals are due to impaired neurogenesis, by changing systematically the location of the familiar and novel objects between the trials we aimed to reinforce the learning hippocampal-dependent component of the NOR test and, consequently, the induction of neurogenesis. Actually, a large body of experimental evidence indicates that the hippocampus plays a critical role in spatial memory^36^,^37^, that learning increases the production of new hippocampal neurons^31^,^32^,^33^, and that during NOR this brain structure is involved in the identification of spatial changes in object position and to the detection of novelty^38^. We also searched to model pattern separation. This is a term derived from computational models of hippocampal function that designates the process that maps similar input patterns to dissimilar representations^39^,^40^. At the cell population level, pattern separation is assessed by measuring the firing rate of hippocampal neurons in animals exposed to similar or interfering inputs whereas in behavioural experiments pattern separation is inferred from the ability of an animal to distinguish very similar stimuli which are closely presented in space and/or time. Actually, it has been shown that the firing rates of the hippocampal neurons recruited by the exposure to a given environment, change when minor modifications are made to the environment whereas an independent set of hippocampal neurons is recruited when more substantial changes to the environment are introduced, i.e. recording in a different room^41^,^42^. Accordingly, behavioural tasks in which the animal needs to discriminate two very similar environments or two very closed visual stimuli to get a food reward^43^,^44^, or in which the animal responds to two similar contexts by conditioned fear^45^ are widely accepted as correlates of pattern separation. Likewise, the capacity of an animal to determine whether one among three identical objects presented during the sample phase is in the same position or was moved to a new location close to the position it occupied before, is considered a behavioural expression of pattern separation^34^,^46^ Indeed, these protocols have been used to determine the role of neurogenesis on pattern separation^44^,^45^, or to demonstrate the role of BDNF in pattern separation^34^, though the firing rate of hippocampal neurons was not measured during the task. In contrasts to these tests, which relay on a change of context or on the short distance between the objects as the source of interference for pattern separation, in the behavioural paradigm presented here, the spatial and the object recognition components of learning are used in combination as interference. We reasoned that the repeated exposure to a familiar object at different places within a familiar context along with the presentation of a novel object (Fig. 2a), calls upon similar demands on pattern separation processes as in the previously described tasks because it evaluates the capacity of the animal to discriminate and encode into distinct memory representations information that is very similar and easily confused for one another and, at the same time, reinforces the memory updating component of the test, allowing past episodes of object recognition to be reactivated, interleaved with new experience and consolidated.

Using this learning paradigm, control animals spent more time exploring the familiar object during the test sessions than during the sample phase but, concurrently, exhibited a clear preference to explore the novel object. Indeed, the total amount of time spent by each rat exploring the objects increased progressively from one test trial to the other. However, this increase in exploration was always made in favour of the new object. The enhanced level of exploration of the displaced familiar object during the testing sessions in relation to the sample phase, reflects the fact that the animal identifies the new position of the familiar object, and so, has integrated the notion of what and where, while the preferential exploring of the novel versus the familiar object indicates that the rat makes a clear distinction between an object encountered before and the novel one and, consequently, has integrated the concept of what as well as the temporal order in which the objects were presented. We suggest that this exploratory behaviour corresponds to the behavioural expression of episodic-like memory and models the pattern separation and consolidation/reconsolidation components of memory because it reveals not only the capacity of the animal to recognise the familiar object (the critical memory), and discriminate where it was previously located but also its faculty to integrate, during the process of remembering, new highly confusable information on a novel object.

Our experimental design is similar to the one used by Haettig *et al.*^47^. Like in the present study, the authors moved the familiar object to a new location and placed a novel object in the arena during the test session. In contrast to our observations, however, animals were unable to distinguish between the familiar and the novel objects 24h after the sample session. We believe that these discrepancies are explained by the absence of a reconsolidation session in the protocol used by Haetting *et al.*, Indeed, whereas in the present study long-term memory (24h) was evaluated after a short-term (2h) memory session that served to reconsolidate the memory trace, in Haetting *et al.* long-term memory was analysed after a single sample session.

When the learning performances of control and PR rats were compared, we noticed that 2h after the sample session, the offspring born to protein-restricted dams were unable to discriminate the novel and the familiar object while their control counterparts clearly differentiated the two objects. During the second test session, performed 24 h after the sample phase, control and PR animals exhibited the same learning performance. Nevertheless, seven days after the sample session PR rats failed again to discriminate between the familiar and the novel objects. The additional test session required by PR rats to distinguishing between the novel object and the familiar one, clearly indicates that early malnutrition impairs the initial encoding and acquisition of the object trace whereas the failure to differentiate the novel object from the familiar one during the third test session, might be the expression of either impaired memory consolidation or impaired memory retrieval. We favour the former interpretation because PR rats were able to distinguish the two objects during the second learning session demonstrating that they have successfully encoded and retrieved the trace.

There were no differences between control and PR rats in the total amount of time they spent exploring the objects, neither during the sample phase nor during the NOR sessions, indicating that the impaired learning performance exhibited by PR animals during the first and third test sessions is not due to an attention deficit but likely results from impaired pattern separation. That is, from their incapability to discriminate and separate into distinct memory representations the displaced familiar object and the novel one. Moreover, control and PR animals showed identical levels of locomotor activity as indicated by the similar number of line crossings during the habituation and test sessions. Given that, in rodents, altered spontaneous locomotor activity in an open field is considered a behavioural expression of anxiety^48^, this latter observation indicates that the level of emotional reactivity of malnourished animals is not altered under our testing conditions and, consequently, that their cognitive deficits result from the impairment of learning and memory and not from the interference of stress with these processes.

Once encoded, the memory trace enters into a labile state to be converted to a permanent one through a consolidation process that takes hours (synaptic consolidation), or weeks to be accomplished (systems consolidation). During this period, the transformation of short-term memory into long-term memory can be disrupted by the addition of new information to the memory trace^49^. Moreover, consolidated memories become unstable and sensitive to disruption upon recall such that a further consolidation process, termed reconsolidation, is required for the trace to be stored in the long term^50^. The herein modified version of the NOR test is based on the acquisition of new learning during memory retrieval and, thus, our behavioural data do not allow to clearly determine whether the cognitive deficits presented by PR rats are due to incomplete consolidation or result from impaired reconsolidation. However, though consolidation and reconsolidation processes share some molecular and neuronal pathways, they are also underpinned by distinct molecular mechanisms. In particular, consolidation, but no reconsolidation, relies on the expression of BDNF^34^. Conversely, the transcription factor Zif268 mediates the reconsolidation but not the consolidation process^35^,^51^. Notably, BDNF and Zif268 mRNA expression are rapidly and transiently up regulated in response to learning. In both cases, the peak in expression occurs at 1h after the exposure to the learning task with a progressive return to basal levels at 90–120 min^52^,^53^. In addition, it has been shown that blocking the expression of BDNF in the hippocampus before the memory task or within the first 6 hours after learning, impairs pattern separation and the consolidation of memory^34^,^52^. These observations clearly indicate that the increased expression of these factors acts as the triggering stimulus for memory formation and consolidation and that their action is limited to the first hours of the learning process. On the basis of these data, we first assessed the expression of BDNF and Zif268 in naive rats and evaluated the effect of learning on the mRNA levels of these factors at the 2 h temporal point.

Protein-restriction reduced the basal levels of BDNF mRNA in the hippocampus without altering the gene expression of Zif268. Moreover, whereas the NOR task increased the expression of both BDNF and Zif268 in control rats, only the mRNA levels of Zif268 were enhanced by NOR in PR rats. On the basis of this observation, we believe that the cognitive deficits exhibited by early-malnourished animals are mainly, if not exclusively, due to impaired consolidation.

Several studies have previously shown that reducing hippocampal neurogenesis impairs pattern separation^44^,^54^ and that improved pattern separation is associated with^55^ and stimulated by^45^ the production of new hippocampal neurons. We therefore examined whether the cognitive defects of PR rats could be associated with reduced hippocampal neurogenesis. The results of these experiments showed that early protein-restriction decreases cell proliferation as indicated by the 22% reduction in the number of hippocampal PCNA immuno-labelled cells in PR rats as compared with controls. There was also a 30% decrease in the number of BrdU-positive cells in the hippocampus of PR rats. This latter observation, contrasts with the results of a previous study in which we observed that the number of BrdU cells in adult rats that were exposed to multi-calorie restriction during early life was reduced by 65%^28^. This discrepancy might be explained by the different nutritional insult as well as by the differences in the temporal analysis of BrdU labelling. Indeed, in our previous study, a consistent reduction in the number of cell proliferation was observed when BrdU labelling was analysed 2h after a single pulse of BrdU in adult rats that were undernourished during gestation and lactation or during the lactation period only. In contrast, only adult animals undernourished during gestation and lactation exhibited a decreased number of BrdU-labelled cells three weeks after the single injection of BrdU^28^.

Importantly, we observed that control and early malnourished rats exhibit the same proportion of BrdU/NeuN co-labelled cells in relation to the total number of BrdU immune-positive cells indicating that perinatal malnutrition does not interfere with the differentiation into neurons of the newly generated cells. Nonetheless, consistent with the decreased rate of cell proliferation, the absolute number of new neurons is significantly reduced in PR rats as compared to controls. This observation is in agreement with our previous finding in the hippocampus of adult offspring born to calorie-restricted dams^28^. Moreover, whereas control rats exhibited a significant increase in the number of both BrdU and BrdU/NeuN co-labelled cells in response to NOR, no significant differences in cell number nor in the number of new neurons were observed between naive undernourished animals and PR rats exposed to the NOR task. These results demonstrate that early protein-restriction impairs the capacity to produce new hippocampal neurons both under basal conditions and in response to a learning task. These data provide also compelling evidence that the long-term impairment in pattern separation and memory consolidation exhibited by early protein-restricted rats is likely due to reduced hippocampal neurogenesis. However, though our observations are formally identical to the results of previous studies showing a link between the reduction of adult neurogeneis induced by focal-x irradiation^44^ or by chemical and genetic methods^56^, and impaired pattern separation, further studies are necessary to firmly establish a cause-and-effect relationship between the herein described decreased production of new hippocampal neurons and the impaired coding of pattern separated memories in early malnourished rats.

All the previous studies aimed at determining the impact of nutritional deprivation during early life on memory have relied on the use of cognitive models that include a reinforcing stimulus or an emotional factor^4^. However, early malnutrition produces enduring changes in emotional reactivity and/or motivation. Consequently, it is not known with certainty whether the impaired learning behaviour exhibited by early-malnourished animals is due to altered motivational or emotional responses to the leaning tests, rather than to a real cognitive deficit. The NOR test does not involve positive or negative reinforces (e.g. food, electric shocks), and requires a minimum of motivational manipulations. Moreover, there were no differences in emotional reactivity between control and PR rats during the NOR test under our experimental conditions. Therefore, our results strongly suggest the existence of a causal relationship between malnutrition in early-life and impaired learning in adulthood.

The long term effects of early nutrition, as well as those of other early and transient environmental stimuli, are thought to be underpinned by epigenetic mechanisms. That is, by the regulation of heritable modifications in gene expression through biological processes that induce a change in chromatin structure without altering the DNA sequence. Epigenetic mechanisms include DNA methylation and histone modification and, indeed, a large body of experimental evidence accumulated during the last years has shown that the adult offspring born to nutrient-restricted dams exhibit a different DNA methylation profile and an altered pattern of histone modifications^57^. There is also strong supporting evidence that epigenetic mechanisms are at the heart of learning and memory processes and play an important role in the regulation of adult neurogenesis^58^,^59^. Therefore, the cognitive deficits, as well as the reduced production of new hippocampal neurons, exhibited by protein-restricted rats, might be underpinned by epigenetic modifications. In support of this idea, we have observed that naive protein-restricted rats exhibit increased acetylation of histone 2B (H2B) and histone 3 (H3) on lysine 9 (H3AcK9) in relation to their control counterparts along with decreased dimethylation of H3 on lysine 9 (H3diMeK9). Moreover, early malnourished rats show less freezing behaviour during fear conditioning and this learning deficit is associated with an impaired posttranslational profile of H3 and H2B in response to the memory test^60^. These data indicate that, in addition to induce stable epigenetic modifications in the hippocampus, perinatal malnutrition alters the plastic epigenetic responses underlying learning and memory.

## Conclusion

We have implemented a behavioural paradigm based on the combination of the novel object recognition and the novel object location tasks allowing to assessing the acquisition of new learning during memory retrieval and the accurate evaluation of episodic-like memory after a single training episode. Using this new behavioural paradigm, we provide compelling experimental evidence showing that maternal protein-restriction induces long-lasting deficits in the encoding and consolidation processes of learning in the offspring and that this latter cognitive defect results from impaired pattern separation and is associated with a reduced production of new adult hippocampal neurons.

## Materials and Methods

### Ethics and Animal use Statment

All experiments were performed in accordance with the European Communities Council Directive of 24 November 1986 (86/609/EEC) and the Principles of laboratory animal care (NIH publication no. 85–23, revised 1985), and were approved by the Ethical Committee for Animal Experimentation of the Pays de la Loire (protocol CEEA.2012.15). All efforts were taken to reduce any animal’s suffering.

### Subjects and nutritional manipulations

Female Wistar rats weighing 200–250 g were obtained from Charles River (St Germain Sur L’arbresle France), and maintained under controlled conditions (12-/12-hour dark/light cycle, 23 ± 1 °C room temperature, food and water ad libitum). After an adaptation period of 10 days, they were mated with male animals of the same strain and age in a proportion of two females per male. Mating was confirmed by the visualization of spermatozoa in a vaginal smear. Pregnant rats were then housed individually and fed either a control (20% protein) or low-protein (8% protein) diet during gestation and lactation. At birth, litter size was adjusted to eight pups per litter with a 1:1 male to female ratio when possible. At weaning, the offspring born to control (C) and protein-restricted (PR) dams were onto standard chow until the end of the experiment. Female pups were discarded from the study to prevent variations due to sex-related differences in learning.

### Learning task and BrdU injections

At the age of three months, control and PR animals were divided into two subgroups of 12 animals each. Naïve animals remained undisturbed in their home cages whereas trained rats were exposed to the learning test which consisted in a habituation phase followed by a sample session for object recognition and three test sessions. The test arena was an open field box (100 × 60 × 40 cm) made of wood with a Plexiglas floor. In order to score line crosses the floor was divided into four squared sections with an indelible marker. During the habituation phase, animals were allowed to freely explore the test arena for 20 minutes every day during 7 days. During these habituation sessions no objects were present in the box. Twenty-four hours after the last habituation session, animals were trained for object recognition by allowing them to explore for 7 min two identical objects placed in the test arena (sample session). Two hours, one day and seven days after the sample session, animals were again placed for 7 min in the test arena to evaluate, respectively, short-term (STM), long-term (LTM) and consolidated memory (CM). During each one of these trials, one copy of the objects used during the sample session was introduced in the test arena along with a novel object. To avoid place preference, objects were counterbalanced and both the familiar and the novel object were placed in a different location from one test session to another and cleaned with 75% ethanol between animals to ensure the absence of olfactory cues. Objects to be discriminated were of different size, shape and colour and were made of plastic or metal material (33 cl soda can bottle). All behavioural sessions were conducted under dim light in a laboratory adjacent to the housing room of the animals and recorded with a video camera affixed to a tripod above the testing area for offline analysis by an investigator blind to the nutritional status of the animals. Object exploration was defined as sniffing or touching the object with the vibrissae or when the animal’s head was oriented toward the object with the nose placed at a distance of less than 2 cm from the object. The time exploring each object was recorded and the capacity to discriminate the novel from the familiar object (discrimination index, DI), was determined by the following formula: DI = [(time exploring the novel object - time exploring the familiar object)/(time exploring both objects)] × 100. A positive DI indicates a preference for the novel object whereas a DI equals to or below zero suggest no recognition. As an index of locomotor activity, the number of line crossings was recorded using as criteria of a line cross when the rat had three limbs cross into de section.

To determine the effects of learning on the production of new hippocampal neurons, naïve and trained animals from both control and PR groups received one i.p. injection of BrdU (150 mg/kg body weight), one hour before each learning test and were sacrificed 2 weeks after the last BrdU administration.

### Tissue processing and immunoflorescence

After deep anaesthesia with sodium pentobarbital, animals were transcardially perfused with phosphate-buffered saline (PBS) followed by 4% paraformaldehyde in PBS. The brains were dissected from the skulls, post-fixed for an additional period of 4 hours in the same fixative and cryoprotected by immersion in 30% sucrose/PBS for 48 hours. Coronal sections (50 μm) through the entire extend of the hippocampus were obtained with the use of a cryostat with every 6^th^ section collected in the same series so that the interval between sections within a given series was 240 μm. Afterwards, sections were stored at −20 °C in a cryoprotectant solution (25% ethylene glycol and 25% glycerine in 0.05M PBS) until processing for immunofluorescence as described previously^28^. In brief, one series of sections of each brain was treated with 50% formamide and 2 × SSC (0.3 M NaCl, 0.03 M sodium citrate), followed by an incubation with 0.1 M boric acid buffer at pH 8.5. After 4 × 5 min washes with PBS, they were incubated in blocking buffer (3% goat serum, 0.3% Triton X-100 in PBS) for 1h and incubated overnight at 4 °C in a mixture of rat anti-BrdU (1:300, Sigma-Aldrich, L’Isle d’Abeau Chesnes, France) plus mouse anti-neuron-specific nuclear protein (NeuN, 1:500; Millipore, Guyancourt, France) antibodies. The next day, sections were washed 4 × 5 min with PBS and exposed to biotinylated anti-rat (1:300, Life Technologies, Villebon sur Yvette, France) for 2 h, and washed 4 × 5 min with PBS. Finally, sections were incubated for 1 h in the dark with strepavidin-conjugated Alexa 568 and with goat anti-mouse Alexa 488 (Life Technologies, Villebon sur Yvette, France). Both secondary antibodies were used at a dilution of 1:500. To ascertain the effects of perinatal protein-restriction on cell proliferation, a second series of sections from each animal was immunolabeled with proliferating cell nuclear antigen (PCNA) using the same protocol and mouse monoclonal anti-PCNA (1:500 from Novastra). Slices were mounted onto slides, and covered under Vectashield (Vector Laboratories).

### Image analysis and neurogenesis quantification

Given the scarcity of BrdU- and PCNA-immunostained cells, the number of new cells was estimated using a modified version of the optical fractionator method. In brief, we employed an exhaustive sampling scheme to count all BrdU-or PCNA-labelled cells on every 12th bilateral section throughout the entire DG between coordinates −2.52 mm at 5.40 mm relative to bregma. Immunostained cells were first visualized with a 40 × objective. The magnification was then increased with a 100 × oil-immersion objective to distinguish single cells. To ensure accurate comparison between groups, we check that the section thickness was the same for all the groups with the aid of a microcator and care was taken to avoid cells in the outermost plane of focus and immune-fluorescence labelled nuclei at the hilar border. The number of BrdU- or PCNA labelled cells per granule cell layer (GCL, including the SGZ) was estimated using the following formula: N = Q × (1/ssf), where Q is the total number of counted cells and 1/ssf is the reciprocal of the section sampling fraction (1/ssf = 12 in the present case).

For the quantification of the double-labelled BrdU/NeuN cells, twelfth bilateral slices per animal spanning the entire hippocampus were used to verify the frequency of BrdU-positive cells expressing NeuN, and six to eight areas per slice were analyzed based on the number of immunostained nuclei and their uniform fluorescence intensity. Eight to twelve optical sections (1 μm thick) were scanned from each area using the 32 × objective. Cells were scored as neurons when the NeuN labeling was unambiguously associated with a BrdU-positive nucleus in stack of sections. Then, the percentages of BrdU-labeled cells that were also labelled with NeuN were calculated for each group.

### Real-time PCR experiments

To investigate the effects of early protein-restriction on the dynamic changes in gene expression induced by learning, an additional set of control and PR animals were trained for object recognition, evaluated for short-term memory and sacrificed by cervical dislocation 2 h after the end of the learning session. Total RNA from the hippocampus was extracted using the TRIzol reagent (Invitrogen) and treated with DNAse (RQ1 DNase, Promega) for 45 min at 37 °C. Later, 1 μg of purified RNA was reverse-transcribed using M-MLV Reverse Transcriptase (Promega) in a total volume of 25 μl, and the resulting complementary DNA was diluted 40-fold in DNAse and RNAse-free water. Thereafter, 5 μl of each complementary DNA diluted sample were used as a template for PCR amplification using primers specific to BDNF exon IV and zif268 and SYBR Green (Qiagen) as a fluorogenic intercalating dye and the iCycler iQ Real-Time PCR Detection System from Bio-Rad Laboratories. Differences in gene expression among the groups were determined using the comparative threshold cycle (Ct) method and 18S RNA as housekeeping gene. The primers sequences are as follows: BDNF forward: TGCGAGTATTACCTCCGCCAT; BDNF reverse: TCACGTGCTCAAAAGTGTCAG; Zif268 forward: TACGAGCACCTGACCACAGA. Zif268 reverse: GGGTAGTTTGGCTGGGATAAC; 18S forward: GATGCGGCGGCGTTATTC; 18S reverse: CTCCTGGTGGTGCCCTTCC.

### Statistical analysis

Experimental results are expressed as means ± SEM. Care was taken to allocate pups of the same mother to different learning conditions (naïve versus NOR), such that the presented results correspond to the combined data from animals born to different dams. Data were analyzed using GraphPad Prism version 5.00 for Windows and were first tested for normality using the D’Agostino & Pearson omnibus normality test for the behavioural data (n = 12 animals per group), or the normal probability plot method for the neurogenesis and gene expression data (n = 6–8 animals per group). In this latter case, we used as criteria of normality an r^2^ value ≥ 0.85 along with the fall of all the data within the 95% confidence intervals of the regression line. Statistical differences between control and PR rats under naïve conditions were assessed by Student’s t-test. The statistical significance of the effects of protein restriction on learning and on locomotor activity were assessed by two-way repeated measures ANOVA using maternal diet and NOR session as independent factors whereas the learning-induced changes on neurogenesis and gene expression were determined by standard two-way ANOVA using maternal diet and NOR as factors. Both analysis were followed by Dunnet’s or Bonferroni’s post-hoc comparisons tests as appropriate. Statistical significance was set at P < 0.05.

## Additional Information

**How to cite this article**: Pérez-García, G. *et al.* Early malnutrition results in long-lasting impairments in pattern-separation for overlapping novel object and novel location memories and reduced hippocampal neurogenesis. *Sci. Rep.* **6**, 21275; doi: 10.1038/srep21275 (2016).
